# Risk factors for major bleeding in the SEATTLE II trial

**DOI:** 10.1177/1358863X16676355

**Published:** 2017-01-31

**Authors:** Immad Sadiq, Samuel Z Goldhaber, Ping-Yu Liu, Gregory Piazza, for the Submassive and Massive Pulmonary Embolism Treatment with Ultrasound AcceleraTed ThromboLysis ThErapy (SEATTLE II) Investigators

**Affiliations:** 1 Vascular Medicine Division, Hartford Hospital, Hartford, CT, USA; 2 Cardiovascular Division, Department of Medicine, Brigham and Women’s Hospital, Harvard Medical School, Boston, MA, USA; 3 Fred Hutchinson Cancer Center, Seattle, WA, USA

**Keywords:** catheter-directed therapy, fibrinolysis, pulmonary embolism, right ventricular function, risk factors, bleeding

## Abstract

Ultrasound-facilitated, catheter-directed, low-dose fibrinolysis minimizes the risk of intracranial bleeding compared with systemic full-dose fibrinolytic therapy for pulmonary embolism (PE). However, major bleeding is nevertheless a potential complication. We analyzed the 150-patient SEATTLE II trial of submassive and massive PE patients to describe those who suffered major bleeding events following ultrasound-facilitated, catheter-directed, low-dose fibrinolysis and to identify risk factors for bleeding. Major bleeding was defined as GUSTO severe/life-threatening or moderate bleeds within 72 hours of initiation of the procedure. Of the 15 patients with major bleeding, four (26.6%) developed access site-related bleeding. Multiple venous access attempts were more frequent in the major bleeding group (27.6% vs 3.6%; *p*<0.001). All patients with major bleeding had femoral vein access for device delivery. Patients who developed major bleeding had a longer intensive care stay (6.8 days vs 4.7 days; *p*=0.004) and longer hospital stay (12.9 days vs 8.4 days; *p*=0.004). The frequency of inferior vena cava filter placement was 40% in patients with major bleeding compared with 13% in those without major bleeding (*p*=0.02). Massive PE (adjusted odds ratio 3.6; 95% confidence interval 1.01–12.9; *p*=0.049) and multiple venous access attempts (adjusted odds ratio 10.09; 95% confidence interval 1.98–51.46; *p*=0.005) were independently associated with an increased risk of major bleeding. In conclusion, strategies for improving venous access should be implemented to reduce the risk of major bleeding associated with ultrasound-facilitated, catheter-directed, low-dose fibrinolysis. **ClinicalTrials.gov** **Identifier: NCT01513759; EKOS Corporation 10.13039/100006522**

## Introduction

Patients with massive and submassive pulmonary embolism (PE) have an increased risk of cardiovascular collapse and death.^1^ Standard anticoagulation alone is inadequate for patients with massive (high-risk) PE.^2^ Early reperfusion therapy with fibrinolysis, surgical embolectomy, or catheter-based ‘pharmacomechanical’ therapy is recommended.^3–5^ For patients with submassive (intermediate-risk) PE, routine reperfusion is not advised. However, a subset of submassive PE patients who have increased cardiac troponin and imaging evidence of right heart dysfunction may benefit from reperfusion.^5,6^ While the data are most extensive for systemic fibrinolysis,^6–8^ clinicians are often hesitant to administer fibrinolytic therapy, even in the highest-risk PE patients, because of the concern of major bleeding, especially intracranial hemorrhage (ICH).

The frequency of major bleeding complications in the setting of fibrinolysis for PE is thought to be related, in part, to the dose of fibrinolytic agent. In 1990, the Food and Drug Administration approved full-dose peripheral venous administration of tissue-plasminogen activator (t-PA) 100 mg over 2 hours for PE. Half-dose fibrinolytic therapy may offer a reduced risk of ICH.^9,10^ Pharmacomechanical catheter-directed fibrinolytic therapy utilizes lower doses of fibrinolytic drug than systemic therapy, and the pharmacological effect is concentrated in the area of greatest thrombotic burden. One of the most extensively studied pharmacomechanical catheter-based techniques is ultrasound-facilitated, catheter-directed, low-dose fibrinolytic therapy, which we evaluated in SEATTLE II for treatment of massive and submassive PE.

SEATTLE II enrolled 150 patients and showed a prompt reduction in the right ventricular (RV)-to-left ventricular (LV) diameter ratio, pulmonary artery systolic pressure, and pulmonary angiographic obstruction at 48 hours without any ICH.^11^ In this exploratory analysis, we evaluate patients who suffered major bleeding events during and after ultrasound-facilitated, catheter-directed, low-dose fibrinolysis to identify risk factors for bleeding.

## Methods

### Study design

SEATTLE II has been reported in detail.^11^ From June 2012 to February 2013, 150 patients were enrolled at 22 sites across the US. Eligible patients were required to have proximal PE (filling defect in at least one main or lobar pulmonary artery), be at least 18 years old, a PE symptom duration of 14 days or fewer, and an RV/LV diameter ratio of at least 0.9 on a contrast-enhanced chest computed tomogram (CT). We included patients with massive (defined as syncope, systemic arterial hypotension, cardiogenic shock, or resuscitated cardiac arrest) or submassive (defined as a normotensive patient with PE and evidence of RV dysfunction) PE. Major exclusion criteria were stroke or transient ischemic attack, head trauma, or other active intracranial or intraspinal disease within 12 months; major surgery within 7 days; recent active bleeding from a major organ; hematocrit less than 30%; and systolic blood pressure less than 80 mmHg despite vasopressor or inotropic support.

### Ultrasound-facilitated, catheter-directed, low-dose fibrinolysis

The EkoSonic^®^ Endovascular System (EKOS, a BTG International Group company, Bothell, WA, USA) procedure was performed by an experienced operator from *Interventional Cardiology, Interventional Radiology*, Vascular Surgery, or Cardiothoracic Surgery. Venous access was obtained, most often with ultrasound guidance, via common femoral or internal jugular venipuncture.

The fixed-dose regimen of t-PA was 24 mg for both unilateral and bilateral PE. A continuous catheter-directed pulmonary artery infusion of t-PA (Genentech, San Francisco, CA, USA) was started at 1 mg/hour for 24 hours for unilateral PE. For patients with bilateral PE, two drug delivery devices were placed, and a continuous infusion of t-PA was started at 1 mg/hour per catheter for 12 hours. No adjunctive interventional techniques to assist thrombus removal or dissolution were permitted. During the procedure, intravenous unfractionated heparin was continued at intermediate intensity with a goal activated partial thromboplastin time (aPTT) of 40–60 seconds. After removal of the catheters, the access site was manually compressed for a minimum of 5 minutes. Fifteen minutes after achieving hemostasis, full therapeutic anticoagulation was reinitiated.

Insertion of inferior vena cava (IVC) filters was discouraged unless the patient developed a contraindication to therapeutic-dose systemic anticoagulation or if the patient suffered recurrent PE despite therapeutic levels of anticoagulation.

### Outcomes

Bleeding complications were assessed for 72 hours following the procedure.

The primary safety outcome was major bleeding within 72 hours of initiation of the procedure. Bleeding events were classified by the Global Utilization of Streptokinase and Tissue Plasminogen Activator for Occluded Coronary Arteries (GUSTO) bleeding criteria.^12^ Major bleeding was defined as either GUSTO moderate or GUSTO severe/life-threatening bleeding events. All monitoring for major bleeding within 72 hours was performed during the hospitalization. All bleeding complications were adjudicated by a designated independent Study Safety Monitor.

### Statistical analysis

Comparisons between those with and without major bleeding were performed by *t*-test for continuous data and by Fisher’s exact test for binary data. Categorical data comparisons with greater than 2×2 dimensions were performed by the Freeman-Halton exact test. Univariate analyses were performed for various baseline and treatment characteristics to assess their relationship with major bleeding status. Co-morbid conditions that could complicate the use of fibrinolytic therapy and had a univariate *p*<0.10 were included in the multivariate logistic regression model as potential predictors for bleeding. These included recent major surgery, cerebrovascular disease, recent gastrointestinal or genitourinary bleeding, and recent trauma. Aside from the multivariate logistic regression analyses, no adjustments for multiple comparisons were made. All analyses were performed using SAS^®^ software version 9.2 (SAS Institute, Cary, NC, USA).

## Results

### The incidence of bleeding

Data from 149 patients are included in this analysis (Table 1) because one patient died from massive PE before completion of the procedure. Fifteen patients (10%) developed 17 major bleeding complications, defined as GUSTO severe/life-threatening and moderate bleeding events (Table 2). None suffered ICH. GUSTO mild bleeding was observed in 20% of patients. GUSTO mild bleeding was observed in 15 (14%) patients with submassive PE and six (25%) of those with massive PE (*p*=0.21).

**Table 1.** Baseline characteristics of patients with and without major bleeding.

	Major bleeding	No major bleeding	*p*-value
	*n*=15	*n*=134	
Mean age ± SD, years	64.6 ± 15.7	58.3 ± 16.2	0.16^a^
Mean body mass index ± SD, kg/m^2^	35.1 ± 8.4	35.6 ± 9.2	0.83^a^
Female, *n* (%)	9 (60)	67 (50)	0.59
Race/ethnicity, *n* (%)			
Caucasian	12 (80)	106 (79)	1.00
African American	2 (13)	20 (15)	
Hispanic	1 (7)	8 (6)	
Hypercholesterolemia, *n* (%)	9 (60)	59 (44)	0.28
Coronary artery disease, *n* (%)	2 (13)	8 (6)	0.27
Hepatic or renal insufficiency, *n* (%)	2 (13)	11 (8)	0.62
Active cancer, *n* (%)	2 (13)	4 (3)	0.11
Heart failure, *n* (%)	1 (7)	6 (4)	0.53
Diabetes, *n* (%)	4 (27)	37 (28)	1.00
Hypertension, *n* (%)	12 (80)	81 (60)	0.17
Tobacco use, *n* (%)	1 (7)	26 (20)	0.31
Obesity, *n* (%)	10 (67)	71 (54)	0.42
Immobility within 30 days of PE, *n* (%)	8 (53)	37 (28)	0.07
Chronic obstructive pulmonary disease, *n* (%)	1 (7)	12 (9)	1.00
History of cancer, *n* (%)	6 (40)	27 (20)	0.10
Family history of venous thromboembolism, *n* (%)	4 (27)	27 (20)	0.52
Major surgery within 30 days of PE, *n* (%)	1 (7)	5 (4)	0.48
Cerebrovascular disease, *n* (%)	0 (0)	1 (1)	1.00
Recent gastrointestinal or genitourinary bleeding, *n* (%)	2 (13)	4 (3)	0.11
Recent trauma, *n* (%)	3 (20)	5 (4)	0.03

a Calculated by *t*-test. All others calculated by Fisher’s exact test.

SD, standard deviation; PE, pulmonary embolism.

**Table 2.** Summary of Global Utilization of Streptokinase and Tissue Plasminogen Activator for Occluded Coronary Arteries (GUSTO) severe/life-threatening and moderate bleeds.

Bleeding event	Site of bleed	Transfused blood products	Hemoglobin (g/dl) or hematocrit (%) (from baseline to lowest)	Complications and outcome
Access site hematoma	Right groin	2 units PRBCs	HCT: 36.1 to 25.2	Transient hypotension requiring vasopressor support; recovered
Access site hematoma	Right groin	2 units PRBCs	Hgb: 13.3 to 7.5	Recovered
Access site pseudoaneurysm	Right groin	2 units PRBCs	HCT: 34.5 to 23.8	Recovered
Gross hematuria	Genitourinary tract	4 units PRBCs	Hgb: 12.6 to 7.8	Recovered
Mucosal bleeding	Nasal and oropharyngeal	4 units PRBCs	Hgb: 10.7 to 7.5	Recovered
Hematoma	Left arm	6 units PRBCs	Hgb: 13.6 to 8.1	Recovered
Hemoptysis	Pulmonary	3 units PRBCs	Hgb: 16.5 to 8.9	Patient was intubated and ventilated; bronchoscopies were performed; recovered
Anemia	Unclear	4 units PRBCs	HCT: 38.8 to 23.2	Recovered
Hematoma	Scrotal surgery site (penile implant)	4 units PRBCs	Hgb: 14.6 to 7.5	Recovered
Hematoma	Abdominal surgery site (hysterectomy)	4 units PRBCs	HCT: 32.1 to 23.9	Recovered
Anemia	Unclear	1 unit PRBCs	Hgb: 11.3 to 9.8	Recovered
Access site hematoma	Right groin	2 units PRBCs	HCT: 37.5 to 25.2	Recovered
Hematoma	Chest wall	2 unit PRBCs	HCT: 43.8 to 29.7	Recovered after prolonged hospitalization
Hematoma	Retroperitoneal	2 unit PRBCs	HCT: 43.8 to 29.7	Recovered after prolonged hospitalization
Hematoma	Chest wall	9 units PRBCs	HCT: 42.9 to 23.4	Recovered after prolonged hospitalization
Hemoptysis	Pulmonary	2 units PRBCs	HCT: 33.8 to 26.3	Died due to pulmonary embolism
Anemia	Unclear	2 units PRBCs	Hgb: 11.7 to 8.2	Recovered

HCT, hematocrit; Hgb, hemoglobin; PRBCs, packed red blood cells.

### Sites of major bleeding

**Figure 1.**
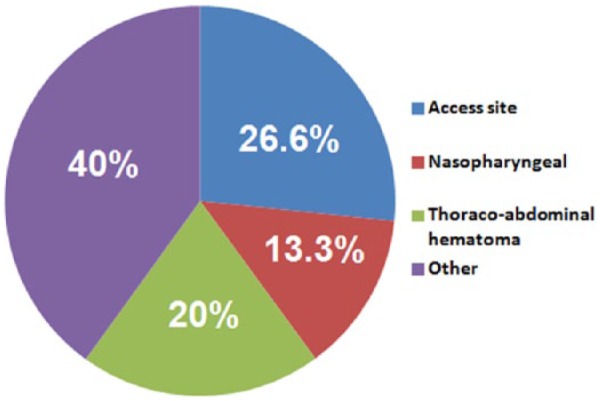
Frequency (%) of location of major bleeding events.

Of the 15 patients with major bleeding, four (26.6%) developed access site-related bleeding (Figure 1). Three patients (20%) developed thoraco-abdominal wall hematomas, while two (13.3%) suffered nasopharyngeal bleeding. One patient developed major bleeding from the site of hysterectomy and required blood transfusion.

### Relationship between baseline characteristics and major bleeding

No significant differences were noted with regard to age, sex, race, body mass index (BMI), and most baseline co-morbidities between those who developed major bleeding and those who did not (Table 1).

### Major bleeding and dose of t-PA and heparin

The dose of t-PA administered in both groups was identical, with a median dose of 24 mg (Table 3). The median duration of fibrinolytic therapy in both groups was 12 hours. The mean aPTT was similar in both groups (40.8 ± 18.5 seconds for major bleeding vs 38.5 ± 26.8 seconds for no major bleeding; *p*=0.8).

**Table 3.** Characteristics of medications administered during the study.

	Major bleeding	No major bleeding	*p*-value
	*n*=15	*n*=134	
Any pre-study anticoagulant/antiplatelet medication, *n* (%)	13 (87)	112 (84)	1.0^a^
Mean cumulative heparin dose administered during procedure ± SD, units	70,463 ± 52,279	58,611 ± 58,221	0.42
Mean aPTT ± SD, seconds	40.8 ± 18.5	38.5 ± 26.8	0.83
Mean total tissue plasminogen activator dose administered during procedure ± SD, mg	23.3 ± 2.4	24 ± 2.1	0.35
Mean duration of total tissue plasminogen infusion during procedure ± SD, hours	12.4 ± 3.3	12.9 ± 2.3	0.29

a Calculated by Fisher’s exact test. All others calculated by *t*-test.

SD, standard deviation; aPTT, activated partial thromboplastin time.

### Major bleeding and burden of PE

An analysis of PE severity showed that more patients had massive PE in the major bleeding group than in the group without major bleeding (47% vs 18%; *p*=0.02).

### Major bleeding and vascular access

Most patients in the study (87%) received two devices for delivery of fibrinolytic therapy and hence required more than one site of vascular access (Table 4). However, there was no difference in the number of devices used between those who developed major bleeding and those who did not. The majority of devices (238/278; 86%) were inserted via femoral venipuncture. Of these, 177 (74%) were inserted via the right femoral vein. All patients with major bleeding had femoral vein access for device delivery, compared to 84% in the group without major bleeding (*p*=0.04).

**Table 4.** Characterictics of vascular access and catheter placement.

	Major bleeding	No major bleeding	*p*-value
	*n*=15	*n*=134	
Number of catheters per patient, *n* (%)			0.69
1	1 (7)	19 (14)	
2	14 (93)	115 (86)	
Access site, *n* (%)			0.04^b^
Right femoral	25 (86)	152 (61)	
Right jugular	0 (0)	31 (12)	
Left femoral	4 (14)	57 (23)	
Left jugular	0 (0)	0 (0)	
Other site	0 (0)	9 (4)	
Catheter placement attempts, *n* (%)			<0.001^b^
1	21 (72.4)	240 (96)	
2–3	7 (24.1)	9 (4)	
>3	1 (3.5)	0 (0)	
Ultrasound-guided vascular access^a^, *n* (%)	22 (76)	180 (72)	0.83

a *n*=29 attempts for patients with major bleeding and *n*=249 attempts for patients with no major bleeding.

b Calculated by Freeman-Halton exact test. All others calculated by Fisher’s exact test.

The use of ultrasound guidance for obtaining venous access to deliver therapy was common (73%). There was no difference in the utilization of ultrasound guidance between the two groups; however, multiple venous access attempts were more frequent in the major bleeding group (27.6% vs 3.6%; *p*<0.001).

### Major bleeding and use of inferior vena cava filter

The overall rate of IVC filter insertion in the study was 16%. The frequency of IVC filter placement was 40% in the major bleeding group compared with 13% in the group without major bleeding (*p*=0.02), and 83% of these filters were placed because of discontinuation of anticoagulation due to major bleeding. IVC filter insertion was performed in 7.4% of patients at the end of an ultrasound-facilitated, catheter-directed, low-dose fibrinolytic procedure through the same sheath and in 8.7% of patients at a later point during the hospitalization as a separate procedure.

### Length of ICU and hospital stay

Patients who developed major bleeding were observed to have a longer length of ICU stay (6.8 days vs 4.7 days; *p*=0.004) as well as a significantly longer length of hospital stay (12.9 days vs 8.4 days; *p*=0.004) (Table 5).

**Table 5.** Length of intensive care unit and hospital stays.

	Major bleeding	No major bleeding	*p*-value^a^
Mean length of intensive care unit stay ± SD, days	6.8 ± 4.8	4.7 ± 3.4	0.004
Mean total length of hospital stay ± SD, days	12.9 ± 7.6	8.4 ± 4.4	0.004

a Calculated by *t*-test.

SD, standard deviation.

### Multivariate predictors of major bleeding

A patient-level multivariate logistic regression analysis was performed to explore potential predictors of major bleeding (Table 6). The variables (univariate *p*<0.10) predicting patients with and without major bleeding were massive versus submassive PE, immobility within 30 days of PE, presence of co-morbid conditions, multiple venous access attempts, use of an adjunctive IVC filter, and baseline serum creatinine level. Only massive PE (adjusted odds ratio 3.6, 95% confidence interval 1.01–12.9; *p*=0.049) and multiple venous access attempts (adjusted odds ratio 10.09, 95% confidence interval 1.98–51.46; *p*=0.005) were independently associated with increased risk of major bleeding.

**Table 6.** Multivariable logistic regression for predictors of major bleeding.

Variable	Major bleeding		No major bleeding	
	Adjusted odds ratio	*p*-value^a^	95% confidence interval	
Massive pulmonary embolism	3.60	0.049	1.01	12.90
Multiple venous access attempts	10.09	0.005	1.98	51.46
Immobility within 30 days of pulmonary embolism	1.94	0.334	0.50	7.50
Any co-morbid condition	3.49	0.095	0.80	15.19
Adjunctive inferior vena cava filter	2.55	0.178	0.65	9.98
Serum creatinine	4.08	0.183	0.52	32.32

a *p*-values by logistic regression.

## Discussion

In SEATTLE II, major bleeding occurred in 10% of patients undergoing ultrasound-facilitated, catheter-directed, low-dose fibrinolysis. Nearly a quarter of these major bleeds were associated with vascular access, including one GUSTO severe/life-threatening bleed. No patient suffered ICH, and there were no fatal bleeds. Factors most strongly associated with an increased risk of bleeding were the presence of massive PE and multiple attempts at obtaining venous access. All patients with major bleeding had femoral access for placement of the ultrasound-facilitated, catheter-directed, low-dose fibrinolysis. Major bleeding was associated with a longer length of hospital stay and more frequent insertion of IVC filters.

Clinical trial and registry data demonstrate that the ‘real world’ use of fibrinolytic agents in PE patients is associated with a high major bleeding and ICH risk. In the International Cooperative Pulmonary Embolism Registry (ICOPER),^13^ the rate of major bleeding was 21.7% and that of ICH 3.0%, while Fiumara et al.^14^ reported a 19.2% and 5.0% frequency, respectively. Previous studies have shown an association between bleeding complications and factors such as older age,^15^ female sex,^16,17^ high or low BMI,^15^ underlying malignancy, and several other co-morbid conditions.^3,18^ In our study, none of these factors significantly influenced the rate of major bleeding. This may have been because of the relatively small number of major bleeding events in this analysis.

We identified multiple access attempts as an area where procedural safety could be improved. Use of ultrasound guidance in obtaining vascular access is becoming widely accepted.^19^ Ultrasound guidance may increase the accuracy of venous access and potentially reduce bleeding complications by minimizing the chance of inadvertent arterial puncture. Routine use of this technique in patients being considered for catheter-directed fibrinolysis may reduce the number of failed venous access attempts. While we found no difference in major bleeding between patients in which venous access was guided by ultrasound and those in which it was not, the relatively small number of major bleeding events may have limited our ability to detect a difference. Other potential strategies to reduce major bleeding complications are to use upper extremity veins (e.g. cephalic) that are not adjacent to an artery and to utilize micro-puncture access needles.

We observed that all patients with major bleeding had femoral access for placement of the ultrasound-facilitated, catheter-directed, low-dose fibrinolysis compared with 84% of those without major bleeding. Femoral access attempts are associated with an increased risk of unintended femoral artery or side branch puncture. Alternative access sites, such as the internal jugular vein, may reduce the risk of major bleeding in patients undergoing ultrasound-facilitated, catheter-directed, low-dose fibrinolysis.

The lack of a comparator group in SEATTLE II precludes any conclusions regarding the relative safety of ultrasound-facilitated, catheter-directed, low-dose fibrinolysis compared with systemic fibrinolysis versus anticoagulation alone. The number of patients enrolled in the study was relatively small and may have limited the power of our multivariate analysis to identify significant predictors of bleeding.

## Conclusions

Massive PE, multiple venous access attempts, and femoral vein access are risk factors for major bleeding associated with ultrasound-facilitated, catheter-directed, low-dose fibrinolysis. These findings point toward continuous quality improvement by focusing on decreasing the morbidity of venipuncture when undertaking catheter-directed thrombolysis.
